# Chronic Dietary Exposure to a Low-Dose Mixture of Genistein and Vinclozolin Modifies the Reproductive Axis, Testis Transcriptome, and Fertility

**DOI:** 10.1289/ehp.0800158

**Published:** 2009-04-01

**Authors:** Florence Eustache, Françoise Mondon, Marie Chantal Canivenc-Lavier, Corinne Lesaffre, Yvonne Fulla, Raymond Berges, Jean Pierre Cravedi, Daniel Vaiman, Jacques Auger

**Affiliations:** 1 Service d’Histologie-Embryologie, Biologie de la Reproduction/CECOS (Centre d’Etude et de Conservation du Sperme Humain), Hôpital Cochin, Paris, France; 2 U567, INSERM (Institut National de la Santé et de la Recherche Médicale), Institut Cochin, Département de Génétique et Développement, Equipe 21 Génomique et Epigénétique de la Pathologie Placentaire, Paris, France; 3 UMR 8104, Centre National de la Recherche Scientifique (CNRS), Institut Cochin, Paris, France; 4 Université Paris-Descartes, Paris, France; 5 Institut National de la Recherché Agronomique (INRA) UMR 1129 FLAVIC and Université de Bourgogne, Dijon, France; 6 Service de Biophysique et Médecine Nucléaire, Hôpital Cochin, Paris, France; 7 INRA/Ecole Nationale Vétérinaire, UMR 1089 Xénobiotiques, Toulouse, France; 8 Département de Génétique Animale, INRA, Jouy-en-Josas, France

**Keywords:** antiandrogen, endocrine disruption, male reproduction, mRNA, phytoestrogen, spermatozoa, toxicology

## Abstract

**Background:**

The reproductive consequences and mechanisms of action of chronic exposure to low-dose endocrine disruptors are poorly understood.

**Objective:**

We assessed the effects of a continuous, low-dose exposure to a phytoestrogen (genistein) and/or an antiandrogenic food contaminant (vinclozolin) on the male reproductive tract and fertility.

**Methods:**

Male rats were exposed by gavage to genistein and vinclozolin from conception to adulthood, alone or in combination, at low doses (1 mg/kg/day) or higher doses (10 and 30 mg/kg/day). We studied a number of standard reproductive toxicology end points and also assessed testicular mRNA expression profiles using long-oligonucleotide microarrays.

**Results:**

The low-dose mixture and high-dose vinclozolin produced the most significant alterations in adults: decreased sperm counts, reduced sperm motion parameters, decreased litter sizes, and increased post implantation loss. Testicular mRNA expression profiles for these exposure conditions were strongly correlated. Functional clustering indicated that many of the genes induced belong to the “neuroactive ligand-receptor interactions” family encompassing several hormonally related actors (e.g., follicle-stimulating hormone and its receptor). All exposure conditions decreased the levels of mRNAs involved in ribosome function, indicating probable decreased protein production.

**Conclusions:**

Our study shows that chronic exposure to a mixture of a dose of a phytoestrogen equivalent to that in the human diet and a low dose—albeit not environmental—of a common anti-androgenic food contaminant may seriously affect the male reproductive tract and fertility.

Estrogenic and antiandrogenic endocrine-disrupting compounds (EDCs) cause a wide spectrum of developmental and fertility detrimental effects [for review, see Sharpe (2003) and Gray et al. (2001), respectively]. Most studies have used high doses of a single compound and short exposure periods, generally during the critical uterine or neonatal period. However, humans and wild animals are exposed simultaneously to various environmental and food EDCs, generally at low levels, throughout their lives. Therefore, it would be valuable to determine the effects of a chronic exposure to low doses of EDCs on the reproductive axis and to identify the mechanisms involved. In particular, it is not known whether lifetime exposures to low (environmental) doses of estrogenic “feminizing” and antiandrogenic “demasculinizing” EDCs can have adverse effects on male reproductive function of the same magnitude as those of acute exposure to high nonenvironmental doses of these compounds in isolation.

In this study, we used a rat model of prolonged exposure to EDCs by gavage to determine the effects on male reproduction of two EDCs that may be associated in the human diet: the phytoestrogen genistein and the antiandrogenic fungicide vinclozolin. Genistein is an estrogenic isoflavonoid found in leguminous plants (Breinholt et al. 2000). It is particularly abundant in diets containing soya or soya-derived products, leading to a dietary exposure of up to 2 mg/kg body weight/day; for infants fed milk formulas containing soya, dietary exposure can reach 1 mg/kg body weight/day (Setchell et al. 1998). In previous studies involving transient gestational, lactational, or adult intakes (Santti et al. 1998), rodents have been exposed to various doses of genistein but the findings are equivocal: some report male reproductive anomalies (Wisniewski et al. 2003), whereas other do not (Faqi et al. 2004; Jung et al. 2004). Human exposure to genistein may affect the responsiveness and sensitivity to other xenobiotics, particularly environmental estrogenic chemicals (You et al. 2002) and other endocrine-active dietary contaminants. Vinclozolin, a dicarboximide fungicide extensively used on fruit and vegetables, is recognized as a human diet contaminant acting—essentially through its two main metabolites M1 and M2—as an androgen-receptor (AR) binding antagonist (Kelce et al. 1994, 1997; Nellemann et al. 2003). A recent French study reported that 20% of 139 meal samples from work canteens contained measurable levels of vinclozolin (Leblanc et al. 2000). In addition, assays of metabolites in urine revealed that > 80% of a population in central Italy was exposed to noticeable levels of vinclozolin and similar pesticides (Turci et al. 2006). Vinclozolin administered to experimental animals *in vivo* at various doses, by various routes, and for exposure periods (gestation, lactation, puberty, adulthood) produces a wide spectrum of reproductive defects: reduced anogenital distance (AGD); persistent nipples; cleft phallus; hypospadias; cryptorchidism; reduced weights of the ventral prostate, seminal vesicles, and epididymis; and reduced sperm counts (Gray et al. 1999; Monosson et al. 1999; Yu et al. 2004). It is highly plausible that vinclozolin can induce such anomalies of the reproductive tract in humans (Kavlock and Cummings 2005). However, most studies used doses 100 times the U.S. Environmental Protection Agency (EPA) no observed adverse effect level (NOAEL) of 1.2 mg/kg body weight/day based on a combination of chronic toxicity, carcinogenicity, and reproductive toxicity in rats (U.S. EPA 2003).

To our knowledge, only one recent study has investigated the reproductive consequences (the frequency of hypospadias) of *in utero* exposure to both genistein and vinclozolin (Vilela et al. 2007). Using a lifelong exposure scheme, we found significant alterations of reproductive development and impairment of several fertility end points by these compounds, the most severe effect resulting from combined exposure to a dietary level of genistein and a level of vinclozolin lower than the U.S. EPA-proposed NOAEL. In addition, we found that mRNA expression profiles in the adult testis are notably and differentially modified according to the exposure protocol. We also describe functional clustering of the genes affected into ontologic families.

## Materials and Methods

### Chemicals

Genistein with a purity of 99% was synthesized at the Laboratoire de Chimie Organique et Organométallique (Université Bordeaux 1, Talence, France). We extracted vinclozolin from the commercial formulation Ronilan (BASF France, Levallois-Perret, France) according to Bursztyka et al. (2008). The extract was dried under vacuum and then recrystallized from methanol. Vinclozolin has a melting point of 108–109°C and its purity, as verified by HPLC-diode-array detection (from 192 to 400 nm) and gas chromatography/mass spectrometry analyses, was > 96% (data not shown). In addition, we tested the absence of the degradation products M1 and M2 by liquid chromatography/mass spectrometry as previously described (Bursztyka et al. 2008).

### Doses used

The exposure scheme consisted of a “high” and a “low” dose for each compound, and the corresponding combinations. The high doses we used were higher than the reported NOAEL of vinclozolin and the plausible levels of genistein in the human diet; we chose these doses to be sufficiently low to maintain normal growth, as well as food and water intake. The genistein high dose, 10 mg/kg body weight (G10), was greater than the genistein levels found in human diets in Southeast Asia (Tanaka et al. 2008) and was several times lower than the doses used in some reproductive studies; the low dose, 1 mg/kg body weight (G1), was similar to that in soya-based diets (Tanaka et al. 2008). The vinclozolin high dose, 30 mg/kg body weight (V30), was substantially greater than real-life exposure levels but was 3–10 times lower than the doses used in several male reproductive studies (Gray et al. 2001). The acceptable daily intake (ADI) of vinclozolin is 600 μg/day/person, corresponding to an exposure of 0.01 mg/kg body weight/day (Food and Agriculture Organization/World Health Organization 1998). In France, the estimated daily intake is 3.3 μg/kg/person (Leblanc et al. 2000), which is < 1% of the ADI. The low vinclozolin dose used in the present study, 1 mg/kg body weight (V1), was higher than human food contamination levels; however, it was lower than the NOAEL combining chronic toxicity, carcinogenicity, and reproductive toxicity in rats (1.2 mg/kg body weight/day; U.S. EPA 2003).

### Animals and regimens

A total of 80 specific-pathogen free (SPF) female and 32 SPF male Wistar Han rats at 8 weeks of age were purchased from Harlan France Sarl (Gannat, France) and fed with a soy-free diet (Harlan Teklad, Gannat, France). On their arrival, they were acclimatized to the animal facility conditions (22°C with constant humidity and a 12-hr light/dark period) for 4 weeks before mating. All animals were treated humanely and with regard for alleviation of suffering. They were maintained in accordance with the French Ministry of Agriculture guidelines for care and use of laboratory animals. From the acclimatization period, animals were fed a purified diet of pellets (18% casein, 46% starch, 23% sucrose, 5% corn oil, 2% cellulose, 5% mineral mixture, and 1% vitamin mixture) and supplied with water *ad libitum*. dissolved in corn oil (Carrefour, Dijon, France) and administered by gavage (2 mL/kg body weight). Control animals were treated with the vehicle alone. Animals were identified by a unique ear tag with a randomly assigned number.

### Exposure conditions

We randomly assigned 10 females to each treatment group and mated them with male rats (five females and two males per cage) with the aim of obtaining at least 20 adult male offspring per treatment group for subsequent data comparisons and analyses. We examined females daily and caged them separately when a vaginal sperm plug was observed [defined as gestational day (GD) 1]. From GD1 until weaning [postnatal day (PND) 21], the dams were gavaged daily with the chemical preparations and then killed under anesthesia. On the day of parturition, all litter sizes were standardized to 10 offspring. From weaning (PND21) to adulthood (PND80), all animals were gavaged daily according to the various exposure protocols, and every 4 days they were weighed and inspected for anomalies.

### Reproductive development end points

On PND25, we randomly selected five males from each group; the AGD was measured with a digital caliper, and the development of the penis was scored according to the procedure reported by Maeda et al. (2000). For both tests, the operator was blind to the treatment group.

### F1 mating and fertility end points

On PND80, six males per group were selected for mating with new unexposed acclimatized females (Harlan France Sarl); each male was housed with one female for 4 days. As described above, females were inspected daily; when a vaginal sperm plug was found, females were housed separately until parturition. We used the following fertility end points: mating index (number of mated females ÷ number of females cohabited with males × 100), fertility index (number of pregnant females ÷ number of mated females × 100), litter size, mean weight per neonate, sex ratio at birth, and percentage of postimplantation loss (number of embryonic scars ÷ number of embryonic buttons and scars × 100).

### Sacrifice

On PND85, we anesthetized males using isoflurane (2.5%) and collected blood under heparin conditions from the dorsal aorta for subsequent hormonal tests. Animals were killed by thoracic cage opening. For each animal, both testes, epididymides and the seminal vesicles, ventral prostate, and liver were excised and trimmed of fat. We then weighed the tissues (Precisa model 125A; Precisa, Poissy, France) and calculated relative weights (weight of the organ in grams per kilogram body weight).

### Sperm motility and motion characteristics

Immediately after epididymis separation, we excised approximately a 0.8-cm portion of the proximal part of the vas deferens and placed it in a Petri dish containing 4 mL of pre-warmed M199 medium (Gibco BRL, Paisley, Scotland) supplemented with 1% bovine serum albumin (Sigma-Aldrich, Saint-Quentin Fallavier, France). After incubating for 5 min at 37°C, we swirled the Petri dish to facilitate the spontaneous release of sperm from the vas deferens. We loaded 10 μL sperm suspension into an 80-μm-deep, 2X-Cel disposable sperm analysis chamber (Hamilton-Thorne Research, Beverly, MA, USA). The device was placed on the heated stage (37°C) of a computer-aided sperm analysis (CASA) integrated visual optical system (Hamilton-Thorne Research). The acquisition parameters and rates used for analysis were those of standard rat analysis setup 1 (frame rate, 60 Hz; frames acquired, 30; minimum cell size, 7 pixels; minimum contrast, 15; brightness, 2,500). Sperm were considered motile if the average path velocity (VAP) exceeded 20 μm/sec and were considered progressively motile when VAP exceeded 50 μm/sec and straightness of trajectory (STR) exceeded 80%. We analyzed a minimum of 200 motile sperm from each animal for the following variables: percentage of motile sperm, percentage of progressively motile sperm, VAP (micrometers per second), straight-line velocity (VSL; micrometers per second), curvilinear velocity (VCL; micrometers per second), lateral head displacement (ALH; micrometers), beat cross frequency (BCF; Hz), STR (%), and linearity of trajectory (LIN; %).

### Epididymal sperm number

The procedure used for assessing the sperm number in the cauda epididymis was based on specific staining of sperm DNA and counting with the CASA instrument (Strader et al. 1996). We dissected out the cauda epididymis just below the point at which the vas deferens joins the epididymis at the distal corpus and at the boundary between the corpus and the proximal end of the cauda epididymis. The tissue was placed in a 50-mL plastic conical tube, chopped finely with scissors, and mixed with 25 mL physiological buffered saline plus 0.05 g/100 mL Triton X-100. The preparation was then crushed with an Ultra Turax T25 basic homogenizer (24,000 rpm 3 × 1 min; IKA, Staufen, Germany) The cauda epididymis preparation was stored frozen at −20°C. On the day of the sperm number assessment, we thawed the preparation, added 0.2-mL aliquots to vials containing the fluorescent DNA-specific dye bisbenzimide (Ident stain; Hamilton-Thorne Research, Danvers, MA, USA), and vortexed it for 30 sec. Following the manufacturer recommendations, we stained the sperm for 2 min. The preparation was vortexed again, immediately loaded into a standard-count disposable 20-μm analysis chamber (Leja, Nieuw-Vennep, the Netherlands), and placed on the CASA stage. The sperm heads were clearly illuminated under an ultra violet beam, identified (and debris ignored), and automatically counted in 10 fields using the RAT-IDENT mode of the CASA instrument. The data are expressed as the total number of spermatozoa per cauda epididymal tissue sample.

### Histology

One testis and one epididymis per animal were fixed in 4% formaldehyde (Sigma-Aldrich), embedded in paraffin, cut into 4 μm-thick sections, stained with hematoxylin and eosin (Sigma-Aldrich), and examined under light microscopy.

### Assays for plasma luteinizing hormone (LH), follicle-stimulating hormone (FSH), testosterone, and estradiol

One plasma sample per animal was frozen at −20°C until assayed. LH and FSH reagents (AH R002 and AH R004, respectively, from Biocode-Hycel) were supplied by OSTEOmedical GmbH (Bünde, Germany). The principle of the rat LH assay is based on competition between the LH of the rat sample and a ^125^I-labeled rat LH tracer for binding to a highly specific rabbit polyclonal antibody. The sensitivity of this assay is 0.14 ng/mL. The rat FSH assay is a solid-phase immunoradiometric assay offering high affinity and specificity for two different epitopes on rat FSH. The sensitivity of the assay is 0.2 ng/mL. We determined total testosterone and estradiol using the COAT-A-COUNT testosterone and estradiol radioimmunoassays (DPC France, La Garenne Colombes, France) according to the manufacturer’s protocols. The detection limits of the assays were 0.2 ng/mL and 2 pg/mL, respectively. All hormone concentrations were determined in duplicate.

### RNA preparation and microarrays

We prepared RNA from individual testes obtained from six animals in each group. RNA was quantified and the quality evaluated using a 2100 Bioanalyzer (Agilent Technologies, Santa Clara, USA). The RNA integrity numbers for all RNAs were > 8. Pools of RNAs from six testes were prepared with strict equilibration of their quantity to ensure equal representation of each individual RNA. After quality control, we sent the samples to the micro array platform of NimbleGen (Reykjavik, Iceland). NimbleGen rat microarrays cover 23,456 transcripts represented by eight 60-mer oligonucleotides spotted in duplicate on the glass slide. The oligonucleotides are isothermic, which allows hybridization at a high temperature acceptable for the complete set of transcripts (70°C). The correlation between the homologous oligonucleotides in each hybridization was > 0.99. We used Cluster 3 (Eisen et al. 1998) and Treeview (Saldanha 2004) for expression clustering, and DAVID software (Dennis et al. 2003) for functional clustering.

### Quantitative reverse-transcriptase polymerase chain reaction (qRT-PCR) validation and interindividual variation

We performed qRT-PCR for 10 genes using cDNA prepared from four individual testes [four animals in each category; PCR primers are described Supplemental Material, Table 2 (doi: 10.1289/ehp.0800158.S1/)]. The reactions were carried out in a 15-μL volume using a Roche Lightcycler (Roche, Meylan, France) and an Invitrogen Sybergreen quantitative PCR kit (Invitrogen, Cergy-Pontoise, France). After 2 min at 50°C and 2 min at 94°C, each reaction was carried out for 40 cycles (94°C for 10 sec, 55°C for 20 sec, and 72°C for 20 sec when fluorescence was measured). This was followed by progressive melting by increasing the temperature from 60°C to 99°C over 10 min, with continuous fluorescence capture. We considered the resulting melting curve satisfactory if one peak only was visible on its derivative. In addition, PCR products were subjected to electrophoresis on an aga-rose gel to check for a single product of the expected size.

### Data analysis

Statistical tests were carried out using BMDP statistical software, version 2 for Windows (Dixon 1988). We determined statistical significance of quantitative differences between treated groups and controls and between treatment modalities using post hoc Tukey’s pairwise comparisons after an analysis of variance. For visual clarity in the tables and figures, we present only the significant differences between each compound or mixture exposure modality and controls. Only pairwise significant differences in the various treatment modalities are reported in text. Differences for qualitative variables between treatment and control groups were tested using the Pearson chi-square test. The significance level is set at 0.05. However, for some aspects of the study (as indicated in tables and figures), we used a threshold of 0.10 because of the relatively small size of the groups. For several figures in both the article and the Supplemental Material (doi: 10.1289/ehp.0800158.S1), we present box plots (rather than standard histograms showing only mean and SE values) in order to present the distributions observed for each exposure group and therefore the true level of variability.

## Results

### Developmental anomalies and body and organ weights

The observed anomalies of the external genitalia are summarized in Table 1. Penile development was immature in all groups except G1, V30, and G10 + V30. Overall, we found the highest rates of genitalia anomalies for the low-dose mixture, G1 + V1, and high-dose vinclozolin, V30. The body weights and relative organ weights in control and treated animals on PND80 are presented in Table 1. The gain in body weight was unaffected by any treatment (data not shown). The relative liver weight was significantly different from control only for G10 (Table 1) and did not vary significantly among the different exposure groups studied. The relative epididymal weight was significantly lower in all treated groups compared with controls (Table 1) and significantly lower in the low-dose mixture (G1 + V1) compared with V1 (*p* < 0.05). Relative testis weights were significantly higher in the high-dose mixture than in the low-dose mixture (*p* < 0.01). The relative prostate and seminal vesicle weights were decreased with V30 compared with controls. In these cases, the addition of genistein had no influence. For these organs, we found no significant difference between all the groups exposed to the compounds.

### Sperm motility and kinematic characteristics

**Figure 1:**
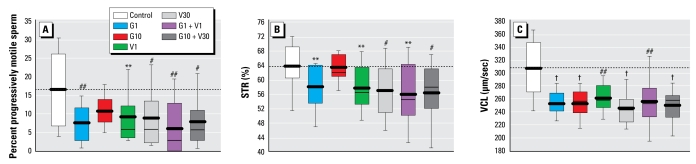
Box plot displaying 10th, 25th, 50th, 75th, and 90th percentile values, and the mean value (thick line) of sperm progressive motility (*A*), STR (*B*), and VCL (*C*) in rats on PND80. **p < 0.05, ^#^*p* < 0.01, ^##^*p* < 0.005, and ^†^*p* < 0.001, compared with controls.

All kinematic characteristics, except for the amplitude of ALH and LIN, were significantly altered in the treated animals. The VCL was significantly lower in all exposure groups than in controls, whereas the percentage of progressively motile sperm and the STR were significantly lower in all treated groups except G10 than in controls [Figure 1; see also Supplemental Material, Table 1 (doi: 10.1289/ehp.0800158.S1)].

### Epididymal sperm number

**Figure 2:**
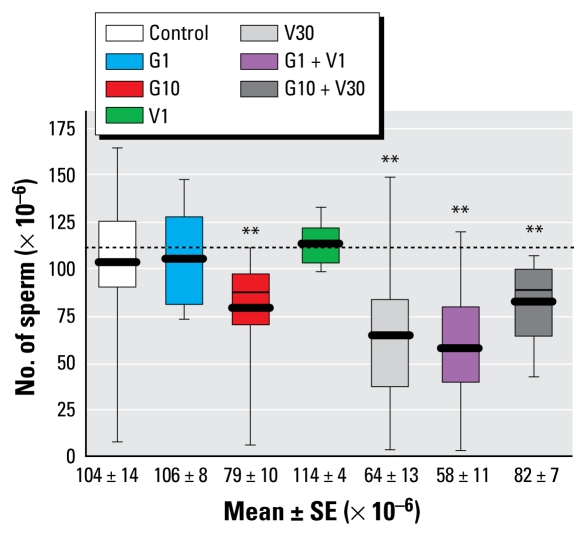
Box plot displaying 10th, 25th, 50th, 75th, and 90th percentile values and the mean value (thick line) of epididymal (cauda) sperm number in rats on PND80. The most pronounced differences in sperm count compared with controls were in the V30 and G1 + V1 groups. **p < 0.05 compared with controls.

The sperm reserve in the cauda epididymis was significantly lower than control values for G10, V30, G1 + V1, and G10 + V30, (Figure 2), and it was significantly lower for the low-dose mixture than for G1 or V1 alone (both *p* < 0.005). We observed the smallest mean value for the low-dose mixture (58 ± 11 vs. 104 ± 14 × 10^6^ spermatozoa in the control; *p* = 0.02), which was not significantly different from that found for V30.

### Histology

Treatment had no obvious effect on testis and epididymis anatomy. However, we observed small variations in tissue/organ histology [see Supplemental Material, Figure 1 (doi: 10.1289/ehp.0800158.S1)].

### Hormone concentrations

On PND80, mean FSH concentrations were significantly higher for G1 + V1, G10 + V30, and V30 than for control groups [13.0 ± 5.1, 11.6 ± 2.0, and 12.3 ± 4.3 vs. 9.9 ± 2.5 ng/mL, respectively; all *p* < 0.05; see Supplemental Material, Figure 2 (doi: 10.1289/ehp.0800158. S1)]. Mean estradiol concentrations were significantly lower than control values only in the G1 + V1 group (3.7 ± 3.9 vs. 6.1 ± 3.6 pg/mL; *p* < 0.05; see Supplemental Material, Figure 2). Testosterone levels were significantly lower than controls in the G10 + V30 and G10 groups (2.0 ± 1.7 and 2.4 ± 2.6 vs. 4.0 ± 3.2 ng/mL; both *p* < 0.05). The LH concentration tended to be higher than control values only in the V30 group (1.34 ± 0.81 vs. 0.97 ± 0.99; *p* = 0.07).

### Mating and fertility

Table 2 summarizes the fertility of the exposed male rats crossed with non exposed females. The mating index was markedly but not significantly lower with the low-dose mixture compared with the control and the other exposure groups. Litter sizes were significantly smaller in the G1 + V1, G10 + V30, G1, and G10 groups; the smallest litters were in the low-dose mixture group (5.3 ± 1.5 vs. 13.0 ± 1.6 in controls; *p* < 0.05). Litter sizes did not differ significantly among the various exposure groups. The postimplantation loss was significantly higher in the V30 and the G10 + V30 groups than in the control group.

### Testis transcriptome analysis

**Figure 3:**
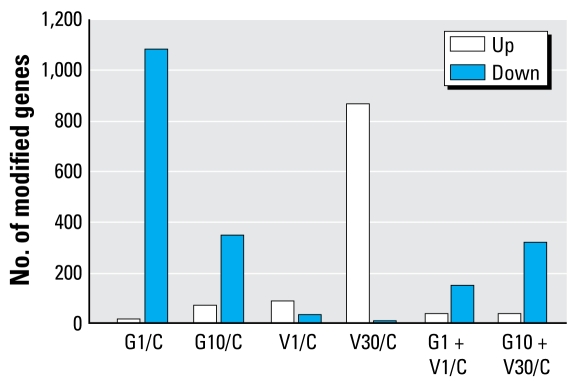
Number of testicular genes modified by the various exposures. C, control. The threshold relative to the controls was > 2.

**Figure 4:**
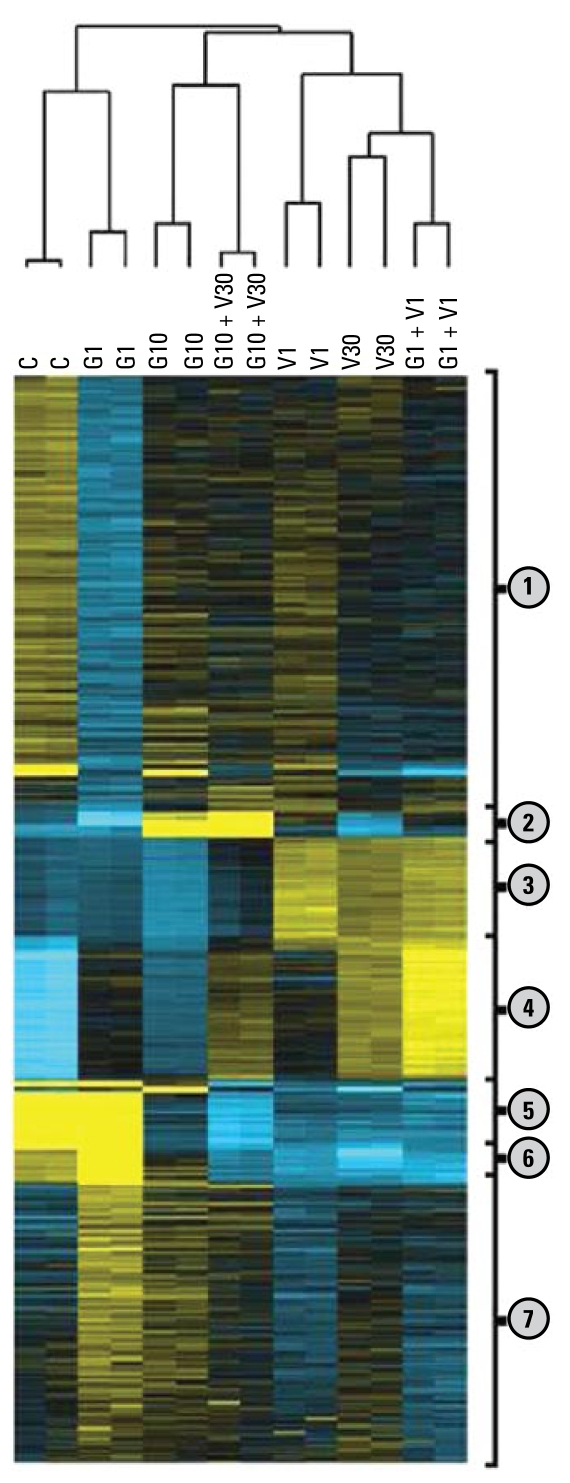
Nonsupervised hierarchical classification revealing seven gene clusters according to the various exposure protocols. C, control. A number of genes were down-regulated by G1 relative to the control (cluster 1). Cluster 2 includes genes induced by G10 and by G10 + V30. Cluster 3 genes were weakly induced by G1 + V1, V1, and V30. Some genes were induced by all the treatments, especially by G1 + V1 and V30 (cluster 4). Cluster 5 contains genes strongly expressed in the control and not modified by G1, all other treatments decreasing their expression. Several genes in cluster 6 were induced specifically by G1. Cluster 7 consists of essentially genes weakly induced by G1 and G10.

The effects of the different treatments on gene repression/induction were strikingly different. Applying a 2-fold threshold, genistein had a generally repressive effect on gene expression in the testis, particularly at the low dose; the high vinclozolin dose (but not the low dose) had the opposite effect (Figure 3). The array results were validated by qRT-PCR with a sample of 10 genes [see Supplemental Material, Table 2 (doi: 10.1289/ehp.0800158.S1)]; consistent with other results obtained with NimbleGen arrays (Buffat et al. 2007), the correlation between the array and the qRT-PCR was > 0.8. Using the complete data set, and no thresholds, the strongest correlation was between G1 + V1 and V30 [*r* = 0.82; see Supplemental Table 3 (doi: 10.1289/ehp.0800158.S1); see also Figure 3]. This is in good accordance with the phenotypic observations: V30 and G1 + V1 had very similar effects on several markers. Nonsupervised hierarchical classification (Figure 4) showed perfectly correlated duplicates and illustrated the clustering of the genes modified by V30 and V1 + G1. This approach identified seven clusters of genes (Figure 4). Functional classification of the genes enabled us to identify biological functions for each sub cluster (Table 3); we functionally analyzed each subgroup of genes using DAVID, after elimination of poorly annotated factors and olfactory receptor genes that are always highly represented in rodent microarrays. Among the genes significantly clustered in KEGG (Kyoto Encyclopedia of Genes and Genomes) pathways, some were involved in regulation of insulin pathways (cluster 1); others were involved in fructose and glucose metabolism (cluster 2). Clusters 3 and 4 comprised genes involved in inter actions between ligands and receptors (e.g., dopamine, acetylcholine, histamine, parathyroid hormone, prostanoids), with the similar composition of the two clusters indicating a quantitative rather than a qualitative effect. The same applies to clusters 5 and 6, which were highly enriched in genes encoding ribosomal proteins. These genes were strongly down-regulated by all treatments except low doses of genistein. This may signify a slowing down of protein synthesis after exposure to the compounds. Finally, cluster 7 was functionally clustered but with relatively low statistical significances, and included mainly genes of the gonadotropin-releasing hormone pathway. We observed the major effect on these genes (up-regulation) in the G1 and G10 groups.

## Discussion

Our study provides evidence that lifelong exposure to low-dose genistein and/or vinclozolin results in a number of anomalies of the male reproductive tract and fertility. This markedly contrasts with previous reports based on short windows of exposure to these compounds, whether gestational, neonatal, or during the puberty/adult periods, showing only mild or no reproductive alterations [see Supplemental Material, Table 4 (doi: 10.1289/ehp.0800158.S1)]. Interestingly, in the adult, despite the absence of macroscopic and microscopic modifications of the testis, we found various changes of the testis transcriptome for all exposure protocols. Furthermore, the intriguing similarities of the phenotypes/genotypes of the animals exposed to high-dose vinclozolin and those exposed to the low-dose mixture, which probably depend on complex modes of action in this chronic exposure from conception to adulthood, may suggest an antiandrogenic action of genistein at a low dose when combined with low-dose vinclozolin (with a possible “synergistic” action because each compound taken alone at a low dose had no or a weak effect). By contrast and for several phenotypes (epididymis, seminal vesicle, and ventral prostate relative weights, as well as litter size), the effect of the high-dose mixture appeared milder than the effect of high-dose vinclozolin alone. This could suggest that, in these specific cases, a “high” dose of genistein could attenuate the effects of vinclozolin. Finally, consistent with these observations, the effects observed for several end points were more pronounced with the low-dose mixture than with the high-dose mixture. Obviously our comments are purely speculative because of the descriptive design of the study, which warrants further work to approach the underlying molecular bases of these intriguing results.

### Phenotype modifications with F_1_ vinclozolin exposures

At a vinclozolin dose of 30 mg/kg body weight, we observed a rate of developmental anomalies of the reproductive tract that are consistent with the higher rate previously reported (Gray et al. 2001); this validates our exposure protocol. At this dose, we also found some of the more detrimental effects on fertility, including a high rate of postimplantation loss (note that this end point was also high for the 1 mg/kg vinclozolin dose). High-dose vinclozolin caused a significant increase in the FSH concentration, suggesting a direct effect on the pituitary axis according to Nellemann et al. (2003). The diminished fertility, evidenced by the reduced litter size, especially with the high dose, could be due, at least in part, to decreased sperm production and motility. It could also be partly the consequence of the marked effects of vinclozolin on sperm chromatin we reported previously (Auger et al. 2004). Prenatal plus infantile exposure of male rabbits to 7.2 mg/kg/day vinclozolin increases the amount of morphologically abnormal spermatozoa, mostly due to nuclear and acrosomal defects (Veeramachaneni et al. 2006). These anomalies may result in impairment of the fertilizing ability, embryonic development, or both (Bartoov et al. 2001; Sailer et al. 1996). Whatever the period of exposure, most published studies involving low-dose vinclozolin report marginal or no effects [see Supplemental Material, Table 4 (doi: 10.1289/ehp.0800158.S1); Colbert et al. 2005; Gray et al. 1999; Hellwig et al. 2000; Shin et al. 2006], contrary to the significant adverse reproductive effects observed at high doses (Yu et al. 2004). Studies of rats exposed to vinclozolin have nevertheless revealed that dose–response relationships are not equivalent among end points (National Toxicology Program 2001). AR expression in the male reproductive system of the rat varies according to time and cell type, such that the effects of vinclozolin may vary substantially according to developmental period(s) and duration of exposure. Indeed, some of the adverse effects observed in adults may depend on changes in AR expression related to the continuous exposure. In this respect, the phenotypic changes we observed with vinclozolin exposure were not necessarily induced only *in utero* or during the early neonatal period. In addition, they could have been mediated by as yet undescribed actions of vinclozolin, or its metabolites, independent of antagonistic action on ARs.

### Phenotype modifications with F_1_ genistein exposures

Chronic exposure to dietary levels of genistein, from conception to adulthood, may have deleterious effects on male reproductive development, adult reproductive organs, and fertility. Some of our findings were in accordance with previous studies: the penis immaturity we observed on PND25 in the G10 group is similar to the delayed preputial separation after gestational and lactational exposure to 5 mg/kg genistein in the diet in Long-Evans rats (Wisniewski et al. 2003) and decreased plasma testosterone (for the “high” dose only) reported by Weber et al. (2001) for a similar dose. This result of Weber et al. (2001) reinforces the idea that genistein decreases the steroidogenic response of Leydig cells, which express both estrogen receptor (ER) α and ERβ, although the expression of these ERs was not affected in our array experiments. Svechnikov et al. (2005) reported that dietary genistein down-regulates an early step of testosterone synthesis (expression of mitochondrial P450 side-chain cleavage enzyme, which catalyses the conversion of cholesterol to pregnenolone). However, as in the study of Roberts et al. (2000), the testosterone levels we found with G1 were not significantly abnormal, suggesting that the effect of genistein on Leydig cells may be dose dependent; consistent with this notion, at the doses and with the strain we used, no difference was detectable in our microarray experiment.

Several of the adverse effects we observed are indisputably related to the estrogen agonist activity of genistein and the localization of both types of ERs that are expressed at various levels in male rat reproductive organs (Boukari et al. 2007; Mäkelä et al. 2000; Pelletier et al. 2000). The lower reproductive performance observed here probably results in part from the combination of sperm movement anomalies for the low- and high-dose exposures and the reduced epididymal reserve (only for G10). Genistein decreases sperm motion variables *in vitro* by inhibiting the sperm protein kinase (Bajpai and Doncel 2003), which our microarray experiments showed to be slightly decreased (reduced to 0.76 for G1). Moreover, genistein has been reported to inhibit α-glucosidase, which is known to sustain sperm motility (Lee and Lee 2001); the expression of this enzyme was also slightly reduced by G1 in our experiment (× 0.78). To our knowledge, the increased postimplantation loss we describe has not previously been reported. However, similar reproductive anomalies have recently been reported for CD-1 mice after neonatal exposure to genistein at environmentally relevant doses (Jefferson et al. 2005), and the reported genotoxicity of genistein may be involved (Stopper et al. 2005). Genistein is a topoisomerase II inhibitor (Markovits et al. 1989), but we did not find any modification of the expression of the corresponding gene in the testis. Another important consequence of the lifelong genistein exposure was the high incidence of reproductive effects, and this contrasts strongly with the absence of effects reported for shorter gestational/lactational, puberty/adult exposures [see Supplemental Material, Table 4 (doi:10.1289/ehp.0800158.S1)]. The possible potentiation of the effects of vinclozolin by exposure to low-dose genistein that we report here might be relevant for human risk assessment because of the similar exposure conditions.

### Phenotype effects of the F_1_ exposures to the mixtures: similarities of the effects with the low-dose mixture and high-dose vinclozolin

In our experimental conditions, the type and magnitude of effects of the low-dose mixture were close to those of high-dose vinclozolin alone. Low combined doses of genistein and vinclozolin significantly increased FSH levels and decreased estradiol levels, an effect very similar to that of high-dose vinclozolin alone. This result parallels the similarities between the effects of the low-dose mixture and the high-dose vinclozolin on sperm production, motility, and kinematics and on the weights of the epididymis and seminal vesicles. These various similarities evoke a synergistic androgenic effect with the low-dose mixture. Genistein has been reported to exhibit *in vitro* antiandrogenic activity in addition to its well-established estrogenic activity (Rosenberg Zand et al. 2000). This type of mechanism may act *in vivo* and contribute to some of the anti androgenic demasculinizing effects observed here with the low-dose mixture, with a magnitude similar to that found for high-dose vinclozolin (the effects found with both exposure modalities, G1 + V1 and V30 were not significantly different for most of the end points studied). Consistent with this notion, combinations of antiandrogens at NOAEL doses have been induced significant synergistic effects *in vivo* (Hass et al. 2007; Kortenkamp 2008). However, more complex modes of actions cannot be ruled out because of our continuous exposure modalities and the multiple biological activities of both the compounds. The complex transcriptomic modifications caused in the adult testis illustrate this. Indeed, although the effect on gene induction of the high vinclozolin dose is generally stronger than that of the combination of genistein and vinclozolin, the overall effect is very similar. As a consequence, the phenotypic alterations may be very similar, even if the effects of the low-dose combination only seldom attain the 2× threshold.

### Testis transcriptome alterations and the phenotypes observed

The gene expression profile under the influence of the EDCs genistein and vinclozolin can be summarized as follows (Figure 4). Genistein administered alone at a low dose had the smallest effects of all treatments tested. The alterations were minor, despite 1,089 genes being down-regulated (using a 2× threshold). This can be explained by the strong and exclusive deregulation of a particular cluster of genes (cluster 4), with most other clusters very similar to the controls. Cluster 4 is composed of genes involved in ligand/receptor interactions, involving numerous hormone peptides and G-protein–coupled receptors. At a higher dose of genistein, with or without high-dose vinclozolin, the alterations were greater. Presumably, the effects of genistein mask the putative effects of vinclozolin, probably indicating that high doses of genistein are the driving force in the gene expression regulation.

The effects of vinclozolin at low and high doses were similar and were strongly correlated with the effects of the low-dose mixture. This suggests that the low genistein dose is able to potentiate the effects of vinclozolin. One possible mechanism is activation by genistein of specific receptors of protein ligands induced by low-dose vinclozolin, and specific preparation of the chromatin to bind specific transcription factors. One of the candidates for such an effect is the gene *Cbx3* (Chromobox, 3 homolog), whose protein has a chromodomain able to regulate chromatin dynamics toward gene induction or repression (Benetti et al. 2007).

## Conclusion

A continuous exposure to low combined doses of genistein and vinclozolin affects male reproductive health by inducing reproductive developmental anomalies, alterations in sperm production and quality, and fertility disorders. The long-term deleterious consequences of chronic exposures to a low-dose mixture of genistein/vinclozolin suggest complex modes of action; we are currently investigating this issue further. Transcriptome analysis may uncover specific factors with potentially major effects on numerous gene targets, such as *Cbx3*.

## Figures and Tables

**Table 1:** Developmental anomalies, incidence of reproductive malformations, and reproductive organ weights in adult rats.

	Treatment						
Observation	Control	G1	G10	V1	V30	G1 + V1	G10 + V30
Development							
Anogenital distance [mean ± SD (mm)]^a^	19.4 ± 0.8	20.0 ± 1.1	19.7 ± 0.9	17.8 ± 0.7*	17.5 ± 0.7*	17.8 ± 0.7*	18.0 ± 1.0
Immature penile development^a^	1/5	1/5	5/5	5/5	2/5	4/5	2/5
Hypospadias (%)	0 (0)	0 (0)	0 (0)	0 (0)	7 (24)	0 (0)	2 (10)
Cryptorchidism (%)	1 (4)	1 (4)	3 (13)	0 (0)	3 (10)	1 (5)	0 (0)
Epididymal anomalies (%)^b^	0 (0)	1 (4)	0 (0)	1 (4)	1 (3)	4 (19)	0 (0)
Other (%)	0 (0)	0 (0)	0 (0)	1 (4)^c^	2 (7)^d^	0 (0)	1 (5)^e^
Overall incidence of anomalies (%)	1 (4)	2 (8)	3 (13)	2 (8)	13 (44)	5 (24)	3 (15)
Organ weight (mean ± SE)							
Body (g)	309 ± 7	350 ± 7#	333 ± 6**	327 ± 8	320 ± 5	322 ± 9	335 ± 6#
Testis^f^	9.7 ± 0.3	9.8 ± 0.2	9.9 ± 0.3	9.9 ± 0.3	10.0 ± 0.3	9.7 ± 0.4	10.6 ± 0.2#
Epididymis^f^	1.56 ± 0.07	1.30 ± 0.03#	1.36 ± 0.07#	1.32 ± 0.05#	1.21 ± 0.09#	1.16 ± 0.06#	1.30 ± 0.04#
Seminal vesicles^f^	3.54 ± 0.26	3.26 ± 0.18	3.24 ± 0.20	3.20 ± 0.15	2.78 ± 0.11#	2.93 ± 0.12**	2.93 ± 0.17**
Ventral prostate^f^	2.28 ± 0.14	2.19 ± 0.09	2.14 ± 0.13	2.08 ± 0.11	1.90 ± 0.07**	2.22 ± 0.14	1.92 ± 0.11**
Liver^f^	30.9 ± 0.7	31.7 ± 0.8	33.1 ± 0.5**	30.8 ± 0.6	31.5 ± 0.7	28.9 ± 0.7*	30.9 ± 0.5

a Measurements and observations on PND25 for five randomly selected animals from each group.

b Macroscopically irregular, cystic, and/or enlarged epididymis.

c Micropenis.

d Two pups from the same litter had multiple anomalies: sexual ambiguity, abnormal seminal vesicles, and absence of vas deferens.

e Sexual ambiguity.

f Relative weights (g/kg).

* Tendency, but not significant (*p* < 0.10).

** *p* < 0.05, and

# *p* < 0.01 compared with controls.

**Table 2:** Effects of treatments on mating and fertility in adult rats at PND80.

	Treatment						
Characteristic	Control	G1	G10	V1	V30	G1 + V1	G10 +V30
Mating index (%)^a^	83	100	83	67	83	50	83
Fertility index (%)^b^	100	100	100	100	80	100	100
Litter size (mean ± SD)	13.0 ± 1.6	7.3 ± 4.0**	7.8 ± 2.9#	11.3 ± 0.5*	7.5 ± 4.1	5.3 ± 1.5**	8.8 ± 2.8**
Pup weight (mean ± SD)	5.6 ± 0.4	6.2 ± 0.7*	6.2 ± 0.6	5.6 ± 0.3	5.9 ± 0.3	6.5 ± 0.4**	6.1 ± 1.1
Sex ratio at birth^c^	0.508	0.591	0.436	0.644	0.663	0.625	0.455
Postimplantation loss (%)	1.5	8.2*	12.5	9.8*	44.4^d^#	10.5*	13.5**

a Number of mated females/number of females × 100.

b Number of pregnant females/number of mated females × 100.

c Total number of males/total number of pups.

d High value mainly due to one female with no pups but 11 implantation sites.

* Tendency, but not significant (*p* < 0.10).

** *p* < 0.05, and

# *p* < 0.01 compared with controls.

**Table 3:** Gene representation and main KEGG pathways identified in the groups defined after the hierarchical clustering of the data from testis cDNA.

Cluster	No. of transcripts	No. of genes	No. of genes(excluding ill-characterized genes)^a^	No. of olfactory-receptor–encoding genes (%)	Main KEGG pathway	*p*-Value	Secondary KEGG pathway	*p*-Value
Group 1	9,401	8,739	5,200	115 (1.2)	Insulin signaling	5.0 × 10^−3^		
Group 2	522	494	369	4 (0.8)	Fructose and mannose metabolism	9.5 × 10^−4^	Neoglucogenesis	1.5 × 10^−3^
Group 3	2,481	2,405	1,422	268 (10.8)	Neuroactive ligand/receptor interaction	5.4 × 10^−13^	Calcium signaling	2.6 × 10^−4^
Group 4	2,801	2,695	1,521	684 (24.4)	Neuroactive ligand/receptor interaction	3.0 × 10^−25^	Calcium signaling	1.6 × 10^−6^
Group 5	1,123	1,076	815	3 (0.3)	Ribosome	4.3 × 10^−14^		
Group 6	469	431	431	4 (0.9)	Ribosome	1.4 × 10^−9^		
Group 7	6,096	5,727	3,788	200 (3.2)	GnRH signaling pathway	1.0 × 10^−3^	Colorectal cancer	4.3 × 10^−3^
							Renin–angiotensin system	1.2 × 10^−2^
							MAPK signaling	1.2 × 10^−2^

Abbreviations: GnRH, gonadotropin-releasing hormone; MAPK, mitogen-activated protein kinase.

a Excluding genes labeled LOC or RGD.
